# Microalgae-based biofertilizer improves fruit yield and controls greenhouse gas emissions in a hawthorn orchard

**DOI:** 10.1371/journal.pone.0307774

**Published:** 2024-08-02

**Authors:** Fen Ma, Yingchun Li, Xue Han, Kuo Li, Mingyue Zhao, Liping Guo, Shifeng Li, Kangjie Wang, Kangxi Qin, Jian Duan, Yutong Liu, Yuxuan Xu

**Affiliations:** 1 Institute of Environment and Sustainable Development in Agriculture, Chinese Academy of Agricultural Sciences, Beijing, China; 2 Shanxi School-Local Cooperative Microalgae Research Institute Co. Ltd., Yuncheng School-Local Cooperative Microalgae Research Institute, Yuncheng, China; 3 Microalgae Resources Agricultural Utilization Laboratory, Yuncheng Difulai Biotechnology Development Co. Ltd., Yuncheng, China; 4 Vegetation Restoration Engineering Technology Research Center, China Agricultural University, Beijing, China; 5 Yuncheng Famous and Excellent Agricultural Products Brand Construction Workstation, Yuncheng Agriculture and Rural Bureau of Shanxi Province, Yuncheng, China; University of Delhi, INDIA

## Abstract

Raising attentions have focused on how to alleviate greenhouse gas (GHG) emissions from orchard system while simultaneously increase fruit production. Microalgae-based biofertilizer represents a promising resource for improving soil fertility and higher productivity. However, the effects of microalgae application more especially live microalgae on GHG emissions are understudied. In this study, fruit yield and quality, GHG emissions, as well as soil organic carbon and nitrogen fractions were examined in a hawthorn orchard, under the effects of live microalgae-based biofertilizer applied at three doses and two modes. Compared with conventional fertilization, microalgae improved hawthorn yield by 15.7%−29.6% with a maximal increment at medium dose by root application, and significantly increased soluble and reducing sugars contents at high dose. While microalgae did not increase GHG emissions except for nitrous oxide at high dose by root application, instead it significantly increased methane uptake by 1.5−2.3 times in root application. In addition, microalgae showed an increasing trend in soil organic carbon content, and significantly increased the contents of soil dissolved organic carbon and microbial biomass carbon, as well as soil ammonium nitrogen and dissolved organic nitrogen at medium dose with root application. Overall, the results indicated that the live microalgae could be used as a green biofertilizer for improving fruit yield without increasing GHG emissions intensity and the comprehensive greenhouse effect, in particular at medium dose with root application. We presume that if lowering chemical fertilizer rates, application of the live microalgae-based biofertilizer may help to reduce nitrous oxide emissions without compromising fruit yield and quality.

## Introduction

Global warming caused by greenhouse gas (GHG) emissions mainly in carbon dioxide (CO_2_), methane (CH_4_) and nitrous oxide (N_2_O), is an important ecological issue, and agricultural cultivation is one of the main sources of GHG emissions [1]. Fertilization is a common agricultural practice to ensure plant yield and quality, meanwhile results in raising GHG emissions [2]. Due to relatively high economic benefits of fruit industry, global fruit planting area has expanded during a recent decade with a global area of 64.9 Mha in 2020 [3]. China is one of the highest fruit producers in the world, with an increase of 42% in orchard acreage from 2000 to 2020 [4]. The expansion of fruit orchards, characterized by high inputs of fertilizer and pesticide, has raised serious concerns over the risk of GHG especially N_2_O emissions [5]. Moreover, Gu et al. [6] reported that N_2_O emissions of orchards were much higher than those of cropland in the same region. For instance, due to high nitrogen fertilizer input, in primary apple-producing area of the China’s Loess Plateau, average annual N_2_O emissions in apple orchard (2.4 kg N_2_O ha^−1^ yr^−1^) were 12% higher than those in wheat field (2.1 kg N_2_O ha^−1^ yr^−1^) [7]. In Taihu region, field measurements also showed that soil N_2_O emissions from peach orchard soils (8.7−26.0 kg N ha^−1^ yr^−1^) were much higher than those measured from rice-wheat system in the same region (0.4−1.1 kg N ha^−1^ yr^−1^) and wheat-maize rotation in the North China Plain (<3.0 kg N ha^−1^ yr^−1^) [8, 9]. As one of the native fruit trees in China, hawthorn has important economic, cultural, and medicine health values. In addition, hawthorn has become Chinese agricultural products with geographical indications, and is of a vital importance in rural revitalization. In 2021, there was an area of 86,700 ha hawthorn planted with a yield of 1.5 Mt [10]. Similar to the major orchard systems such as apple and peach, hawthorn cultivation is associated with high chemical and organic fertilization inputs. Besides, in hawthorn orchards, fertilizer is concentrated around fruit trees and topdressing generally occurs in hot and rainy summers, high N_2_O may be emitted from soils. Furthermore, to the best of our knowledge, no study has examined the GHG emissions in hawthorn orchard fields. Therefore, accurately measuring and mitigating GHG emissions from hawthorn orchard is important for alleviating the pressure of climate change caused by fruit planting growth without affecting the fruit production.

Biofertilizers, containing live microorganisms or bacteria and fungi, have gained prominence as eco-friendly fertilizers, because they can maintain soil fertility and stimulate crop growth [11, 12] while neither affect soil health nor pollute the environment [13]. Microalgae, a highly diverse group of photosynthetic microorganisms including prokaryotes (e.g. cyanobacteria) and eukaryotes (e.g. green algae), have attracted much attentions as a novel type of biofertilizers due to their excellent ability to soil improvement and crop productions [14]. Many studies have been done to indicate the performance of microalgae-based biofertilizer in enhancing growth and yield of staple crops, such as rice [15], wheat [16, 17], maize [18] and potato [19]. On the one hand, microalgae are an input of organic carbon source through photosynthesis and secretion of exopolysaccharides when applied to soil, thus improving soil fertility [17, 20]. Several studies stated that soil organic carbon contents were increased in the soil using microalgae biofertilizers [21–23]. On the other hand, microalgae biofertilizers application could increase soil available nitrogen, phosphorus, and potassium as well as soil microbial activities [24–26]. Microalgae biofertilizers also improve soil fertility and crop yields by facilitating soil aggregation, structure, and stability [22]. Besides, when added to soil as biofertilizers, microalgae can provide phytohormones (e.g. auxins, cytokinins, gibberellins), other bioactive compounds like amino-acids and polyamines, and micronutrients (e.g. Mg, Fe and Mn) to the plant [14, 27]. Moreover, as microalgae can produce a variety of bioactive substances that can influence plant growth through different organs (such as leaves and roots), various concentrations and application modes may have different effects on crop growth. For example, *Chlorella vulgaris* (10%) suspensions increased more quality and yield of Swiss beet in foliar spray than root application [28]. Thus, microalgae biofertilizers could be applied by different methods to maximize effects on promoting plant growth and production.

Compared with the generally positive effects of microalgae biofertilizer on plant growth and soil improvement, the impact of microalgae biofertilizer on GHG emissions is less clear. A handful of studies reported that, soils inoculations with N_2_-fixing cyanobacteria significantly reduced CH_4_ emissions compared to uninoculated soils in rice fields [29–31]. Shrestha et al. [32] found that N_2_O emissions in application of green microalgae biomass were 1.5 to 3-folds lower compared to urea fertilization in a wheat field, and ascribed to overall lower mineral nitrogen availability in soils fertilized by microalgae. However, other studies showed that applying green microalgae biomass significantly increased the emissions of soil N_2_O [33, 34] and CO_2_ [33], but had no significant effect on CH_4_ emissions [33]. Microalgae biofertilizers as organic source are likely to favor the growth of soil microbial populations including nitrifying and denitrifying microbes, and enhance soil respiration [35]. The improved respiration could consume soil oxygen and induce the formation of anaerobic soil microsites that boosting denitrification, resulting in high N_2_O production [36]. In addition, microalgae characterized by high concentrations of macronutrients is expected to favor nitrogen mineralization and nitrification [34]. Increased N_2_O emissions due to applying microalgae biofertilizers may be an undesirable tradeoff. Therefore, it is essential to investigate the effects of microalgae biofertilizers on GHG emissions from agricultural fields, for more comprehensively assessing their applications in agriculture.

In the present study, a field experiment was conducted by applying microalgae biofertilizer (a combination of live cyanobacteria and green microalgae) combined with conventional fertilization to a plant-soil system in a hawthorn orchard. The main objective was to assess the hawthorn yield and quality, GHG emissions, as well as soil organic carbon and nitrogen fractions and soil pH, under the effects of live microalgae-based biofertilizer applied at three doses and two modes. We also aimed to identify the most effective application strategy regarding the live microalgae-based biofertilizer in terms of hawthorn productivity and GHG emissions. Given the background of carbon neutrality target China has issued to achieve by 2060 [37], this study would advance integrated assessment of the environmental and agricultural effects of microalgae application as biofertilizers.

## Materials and methods

### Study site

**Fig 1:**
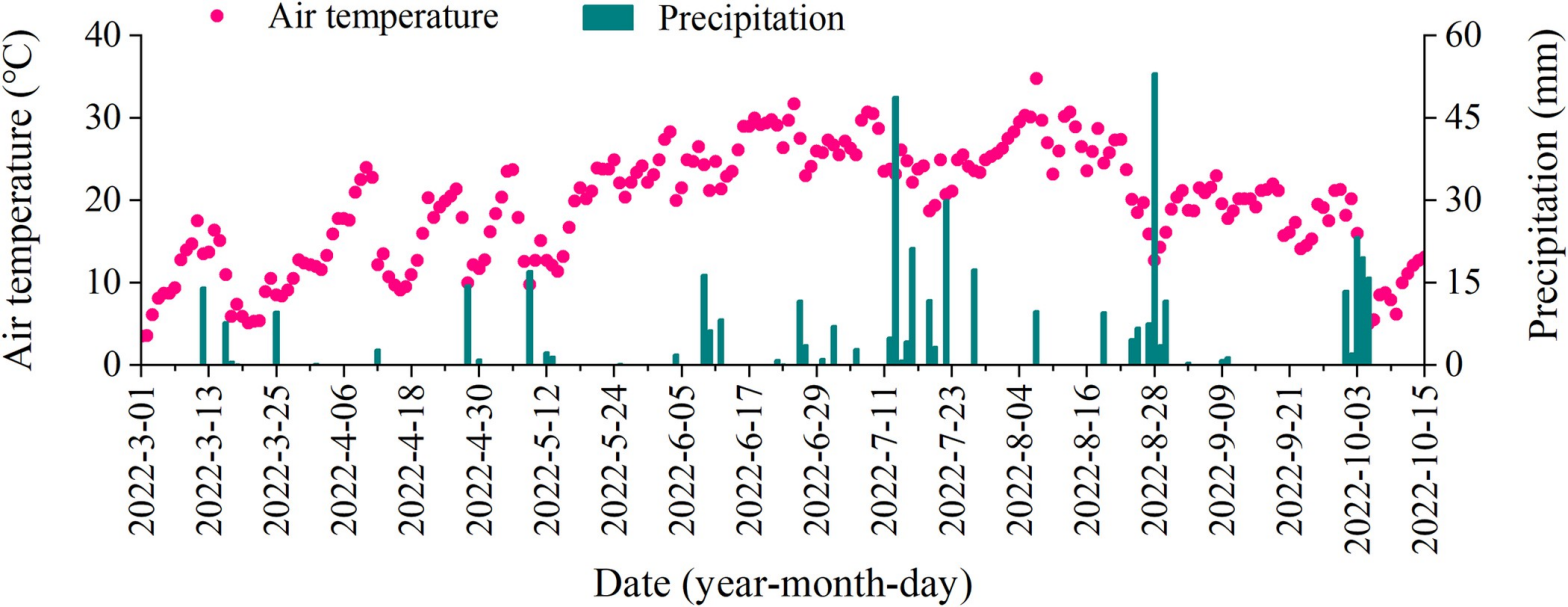
Air temperature and precipitation during the experiment.

The study was conducted at a commercial hawthorn orchard (*Crataegus pinnatifida* Bge.) in Jiangxian (35˚28’48’’ N, 111˚34’12’’ E), Shanxi Province, China. The study area has a temperate continental climate, with an average annual precipitation and temperature of 573.7 mm and 11.9°C, respectively. During the experiment, the total precipitation was 458.9 mm and approximately 60% of that occurred in July and August, and the daily temperature ranged from 3.5 to 34.8°C with an average value of 19.4°C (Fig 1). This was collected from a meteorological station adjacent to the experiment site located at Jiangxian county. The soil is Cinnamon Loess and is defined as a silt loam under the USDA texture classification system. At the beginning of the experiment, the soil properties of 0−20 cm depth were pH of 8.1, bulk density of 1.2 g cm^−3^, soil organic matter of 23.5 g kg^−1^, soil organic carbon of 13.5 g kg^−1^, total N of 1.1 g kg^−1^, alkali-hydrolyzed N of 77.9 mg kg^−1^, available P of 55.1 mg kg^−1^, and available K of 440.4 mg kg^−1^.

### Microalgae strains characterization

The live microalgae-based biofertilizer employed in this study was a combination of cyanobacteria (*Trichormus variabilis* strain) and green microalgae (*Auxenochlorella pyrenoidosa* strain) with a cell density of 8 × 10^6^ cells mL^−1^ [38], respectively, and was provided by Yuncheng Difulai Biotechnology Co., Ltd., Shanxi, China. The microalgae-based biofertilizer has the following properties: chlorophyll content = 4.12 μg mL^−1^ (spectrophotometric method), pH = 7.09 (Mettler-Toledo pH meter), total organic carbon = 49.52 g L^−1^ (automated TOC Analyzer), total nitrogen = 505.55 mg L^−1^ (automated TOC Analyzer), total phosphorus = 12.39 mg L^−1^ (continuous Flow Analyzer).

*Trichormus variabilis* strain belongs to *Anabaena azotica*, with straight or filamentous chains. In nitrogen-rich conditions, the cells on the filament predominantly served as vegetative photosynthetic cells. However, in nitrogen-depleted condition, the vegetative cells differentiate to form heterocysts that are capable of fixing nitrogen. Because heterocysts contain the nitrogenase enzyme of nitrogen fixation, which can use the energy obtained through photosynthesis to reduce dinitrogen to ammonia [39]. In this way, vegetative cells and heterocysts behave with functional division. Specifically, nutrient cells provide carbon source to heteromorphic cells, and in turn heteromorphic cells provide nitrogen source to nutrient cells. The interdependence and interaction between the two cells are necessary to maintain the growth of filament and play a role in agricultural scenario. The *Anabaena azotica* can fix atmospheric N_2_ into plant-available nitrogen and release it through microbial nitrogen mineralization, which promote plant nitrogen uptake and plant growth [40].

*Auxenochlorella pyrenoidosa* strain, belonging to *Chlorella pyrenoidosa*, is a spherical single-cell organism, with round cell body in 3−5 μm diameter and transparent cytoderm. It consists of a cup-shaped chloroplast and a powdered nucleus in intracellular protoplasts. During reproduction, the materials in the cell are divided into small pieces that are round with 0.3−0.7 μm in diameter. Then the larvae scatter out after the maternal cytoderm breaks up. The *Auxenochlorella pyrenoidosa* is also involved in the production of *Chlorella* Growth Factor (CGF), which has the function of promoting cell growth, improving immunity and antioxidant activity [41]. It also can produce large amounts of nicotinamide, thiamine, vitamins B2 and B6, folic acid, inositol, and pantothenic acid. The microalgae biomass contains 57% protein, 2% fat, and 26% carbohydrates, which represent important sources of organic matter for soil microorganisms [27]. The microalgae *Chlorella pyrenoidosa* can be regarded as an accelerator agent in biodegradation of soil organic matter, and thereby aiding in the mineralization and solubilization of nutrients in soil, important for plant growth [42].

### Experimental design

The hawthorn trees (Dajinxing, a popular variety), spaced 4.5 m × 2.5 m (889 trees ha^−1^), were planted in March 2007. The field experiment consisted of seven treatments, each with three replicates in a fully randomized design, giving a total of 21 plots, each with an area of 110 m^2^ (11 m × 10 m). The seven treatments were as follows:

1. CK, conventional fertilization, i.e. compound fertilizer combined with commercial organic fertilizer.
2. L-R, conventional fertilization plus low microalgae dose with root application.
3. M-R, conventional fertilization plus medium microalgae dose with root application.
4. H-R, conventional fertilization plus high microalgae dose with root application.
5. L-RL, conventional fertilization plus low microalgae dose equally divided into root application and foliar spray.
6. M-RL, conventional fertilization plus medium microalgae dose equally divided into root application and foliar spray.
7. H-RL, conventional fertilization plus high microalgae dose equally divided into root application and foliar spray.

Conventional fertilization received the combined application of chemical and organic fertilizers. Compound fertilizer (nitrogen-phosphorus (P_2_O_5_)-potassium (K_2_O): 17-17-17) was separately applied on 6 April (889 kg ha^−1^), 25 June (1334 kg ha^−1^) and 26 August (1334 kg ha^−1^) by surface band application in combination with irrigation of 60 mm approximately. Organic fertilizer (cow manure and crop residues) contained 28.3% organic carbon, 1.4% nitrogen, 1.1% P_2_O_5_, and 1.2% K_2_O, and was applied as basal fertilizer following the previous harvest by band application at about 15 cm depth near the root of hawthorn tree. The microalgae application was undertaken three times on 13 April, 30 June and 31 August at the same rate, 1.0, 2.5, and 5.0 L ha^−1^ for low, medium and high microalgae dose each time, respectively. As for root application of microalgae, a mixture of microalgae and 5 L water per experimental plot was evenly spread over the soil surface within an around 0.5 m radius of the hawthorn tree using a graduated watering can. For foliar spray, a mixture of microalgae and 3 L water per experimental plot was uniformly sprayed on the leaf surface with a graduated watering can. Simultaneously, control plots (single conventional fertilization) were added with the same amount of water as microalgae treatments. The total application rates of conventional fertilization and microalgae were showed in Table 1. Other management measures, including irrigation, pest, and weed control were consistent with local agronomic practices, and kept same for all the treatments.

**Table 1:** Application rates of conventional fertilization and microalgae across treatments.

Treatment	Compound fertilizer (kg ha^−1^)				Organic fertilizer (kg ha^−1^)				Microalgae (L ha^−1^)
	Rate	Specific nutrient input			Rate	Specific nutrient input			
		Nitrogen	Phosphorus (P_2_O_5_)	Potassium (K_2_O)		Nitrogen	Phosphorus (P_2_O_5_)	Potassium (K_2_O)	
CK	3557	605	605	605	3000	42	33	36	0
L-R	3557	605	605	605	3000	42	33	36	3.0
M-R	3557	605	605	605	3000	42	33	36	7.5
H-R	3557	605	605	605	3000	42	33	36	15.0
L-RL	3557	605	605	605	3000	42	33	36	3.0
M-RL	3557	605	605	605	3000	42	33	36	7.5
H-RL	3557	605	605	605	3000	42	33	36	15.0

### GHG emission measurements

In each experimental plot, in-situ GHG fluxes was measured using a static closed chamber method [43], starting from June to September of 2022 when hawthorn showed vigorous growth. Briefly, the static chambers were cylindrical, 30 cm in diameter and 20 cm in height. Permanent rings were inserted into the soil to 10 cm depth as a pedestal for the chambers. A temperature sensor was fixed to the mid-position of each closed chamber to record the air temperature inside.

Gas sampling was undertaken once a week between 9:00 and 11:00 a.m. to minimize the effect of diurnal temperature variation on gas fluxes. During fertilization or rainfall (>10 mm), the sampling intervals decreased to 2 or 3 days. Chambers were closed for 20 min, and gas samples were collected from its headspace at 0 and 20 min respectively. The concentrations of N_2_O, CH_4_ and CO_2_ in gas samples were analyzed using a gas chromatograph (Agilent 7890B, Agilent Technologies Inc., CA, USA) equipped with an electron capture detector (ECD) and a flame ionization detector (FID). Cumulative N_2_O, CH_4_ and CO_2_ emissions were determined by linear interpolation between sampling dates, assuming that the fluxes followed a linear trend during the unmeasured periods [44]. Soil N_2_O, CH_4_ and CO_2_ emissions intensity was calculated by dividing the cumulative N_2_O, CH_4_ and CO_2_ emissions by the hawthorn yield, respectively. In addition, to assess the comprehensive greenhouse effect and hawthorn output at the expense of GHG emissions, GHG emissions were converted to global warming potential (GWP) values and GHG intensity. The GWP was obtained by the sum of cumulative N_2_O, CH_4_ and CO_2_ emissions in CO_2_ equivalents (CO_2_-eq) with conversion factors (on a 100-year time horizon) of 273 and 28 for N_2_O and CH_4_, respectively [1]. The GHG intensity was calculated the CO_2_ equivalents per unit of hawthorn yield [45].

### Hawthorn fruit yield and quality components

In middle October of 2022, the hawthorn fruits were collected manually in each experimental plot. Both the total yield and single fruit weight were determined. Total vitamin C, reduced vitamin C, soluble sugars and reducing sugars were selected, to represent hawthorn fruit quality, determined by molybdenum blue spectrophotometry, 2,6-diohloroindophenol titration, anthrone method and 3,5-dinitrosalicylic acid method, respectively, referring to the Experimental Guidance of Plant Physiology.

This study is complied with relevant institutional, national, and international guidelines and legislation. During the determination of fruit yield and quality components, the collection of hawthorn fruits and used in this study are obtained relevant permissions.

### Soil sampling and measurements

At harvest, five separate soil cores were collected in each experimental plot at 0−10 and 10−20 cm depth, and the soils were thoroughly mixed into a composite sample for each depth, yielding a total of 42 soil samples (7 treatments × 3 replicates × 2 depths). All soil samples were removed visible plant roots with tweezers and passed through a 2-mm sieve, and each soil sample was split into two parts. One part was kept at 4°C immediately, and the other part was air-dried and then stored at room temperature.

Soil organic carbon (SOC) was determined by wet digestion with H_2_SO_4_-K_2_CrO_7_, and total nitrogen (TN) was measured using a C/N element analyzer (Elementar, Germany). The concentrations of ammonium nitrogen (NH_4_^+^−N) and nitrate nitrogen (NO_3_^−^−N) were determined using an AA3 continuous-flow analyzer (Bran + Luebbe Gmbh, Norderstedt, Germany) after extraction with 2 mol L^−1^ KCl in 1:5 soil to solution ratio. Soil microbial biomass carbon (MBC) and microbial biomass nitrogen (MBN) were determined by the fumigation-extraction method [46] and a TOC Analyser (GE Sievers InnovOx, Boulder, USA). In addition, the organic carbon and nitrogen from the non-fumigated soils were considered to be dissolved organic carbon (DOC) and total dissolved nitrogen (TDN), and dissolved organic nitrogen (DON) was calculated as TDN minus NH_4_^+^−N and NO_3_^−^−N. The soil pH was measured using a portable pH meter (Mettler Toledo, Switzerland) with a soil to CaCl_2_ solution volume ratio of 1:2.5.

### Statistical analyses

All data were analyzed using Excel 2016 and the SPSS 20 statistical package for Windows (SPSS China). One-way analysis of variance (ANOVA) with a least significant difference (LSD) test (*P* < 0.05) was used to determine significant differences among treatments in GHG emissions, soil organic carbon and nitrogen fractions as well as soil pH, hawthorn fruit yield and quality. Two-way ANOVA were used to analyze the effects of treatment, sampling date and their interactions on GHG fluxes, as well as the effects of treatment, soil depth and their interactions on soil organic carbon and nitrogen fractions as well as soil pH.

## Results

### Hawthorn fruit yield and quality

**Fig 2:**
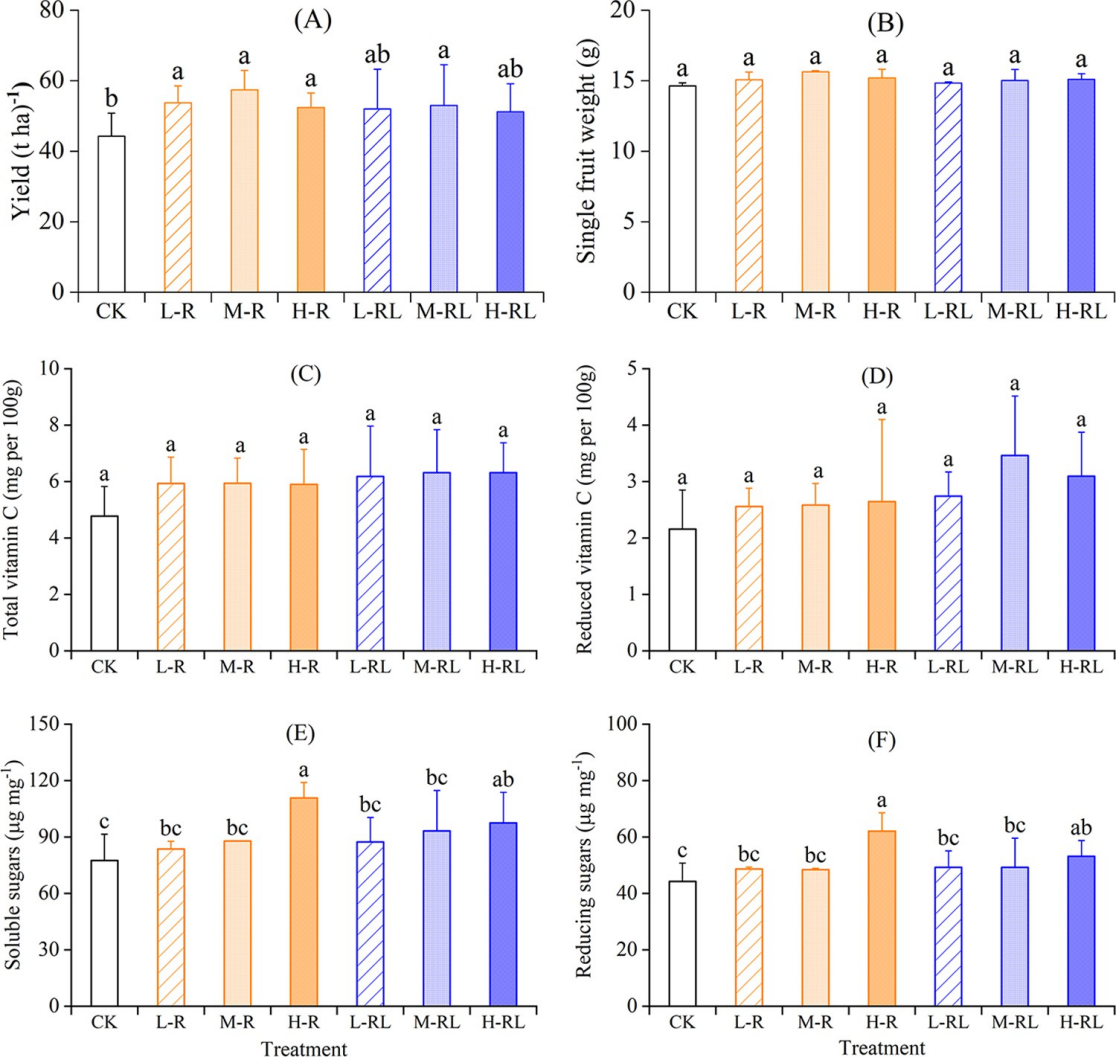
Hawthorn yield (A), single fruit weight (B), total vitamin C (C), reduced vitamin C (D), soluble sugars (E) and reducing sugars (F) under different treatments. Error bars indicate standard deviation of three replicates. Different lowercase letters indicate significant differences among treatments at *P* < 0.05.

The yield of hawthorn, ranging from 44.3 to 57.4 t ha^−1^, was 15.7%−29.6% higher in microalgae application than in CK, with a maximal increment in M-R treatment, while no significant difference was found among different microalgae application doses and modes (Fig 2A). The single fruit weight, which ranged between 14.6 and 15.6 g, was not significantly influenced by microalgae application (Fig 2B).

The contents of total and reduced vitamin C were respectively in the range of 4.8−6.3 and 2.2−3.5 mg per 100 g across treatments, and was not significantly affected by microalgae application (Fig 2C and 2D). The contents of soluble and reducing sugars were respectively in the range of 77.4−110.7 and 44.2−62.1 μg mg^−1^ across treatments, and were significantly increased in H-R and H-RL treatments compared with CK (Fig 2E and 2F).

### GHG emissions

**Fig 3:**
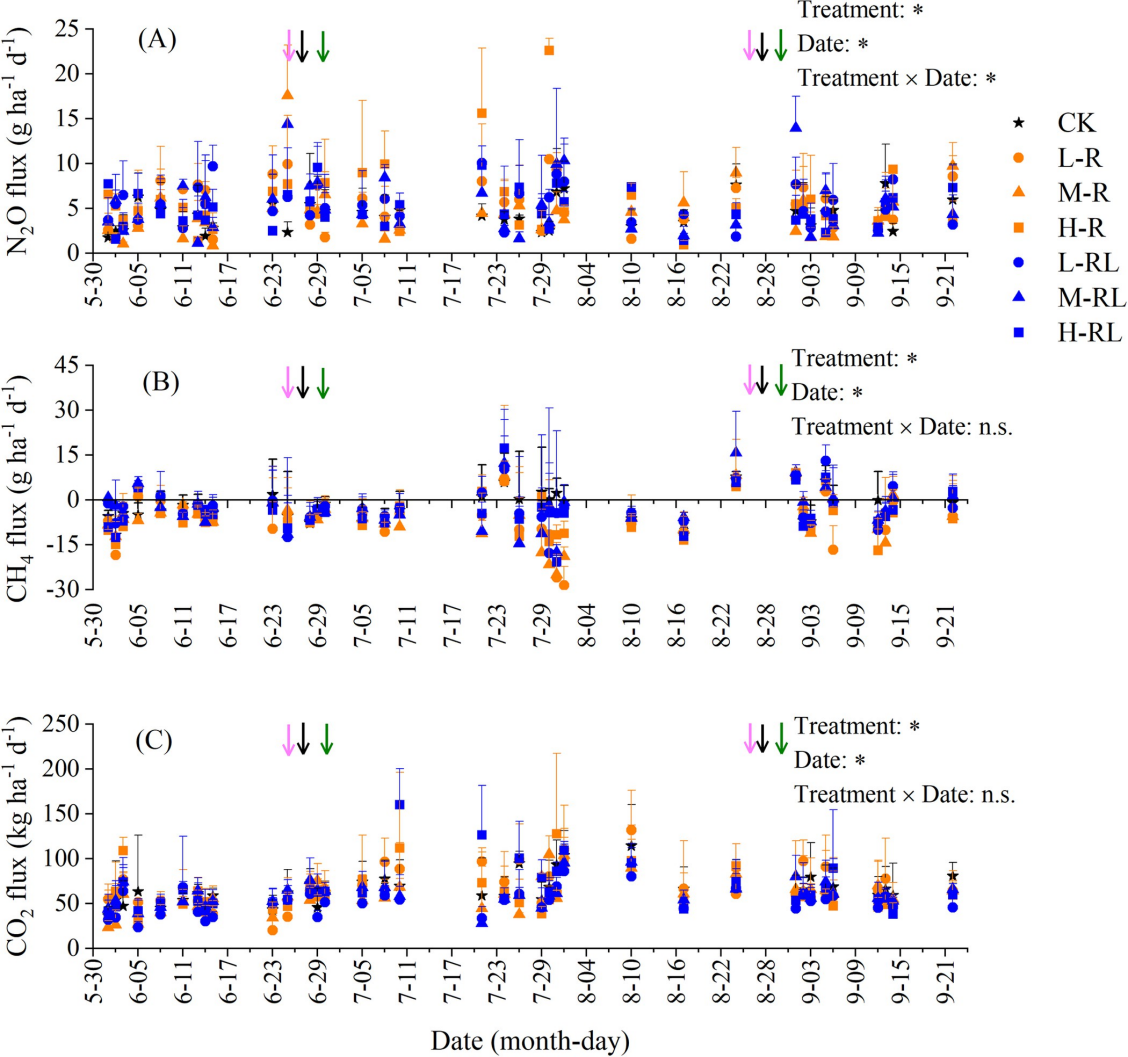
Variation in fluxes of soil N_2_O (A), CH_4_ (B) and CO_2_ (C) from different treatment plots in the hawthorn orchard field from June to September of 2022. The pink, black and green arrows indicate conventional fertilization, irrigation and microalgae application dates, respectively. Error bars indicate standard deviation of three replicates. *: significant at *P* < 0.05; n.s.: no significant.

**Fig 4:**
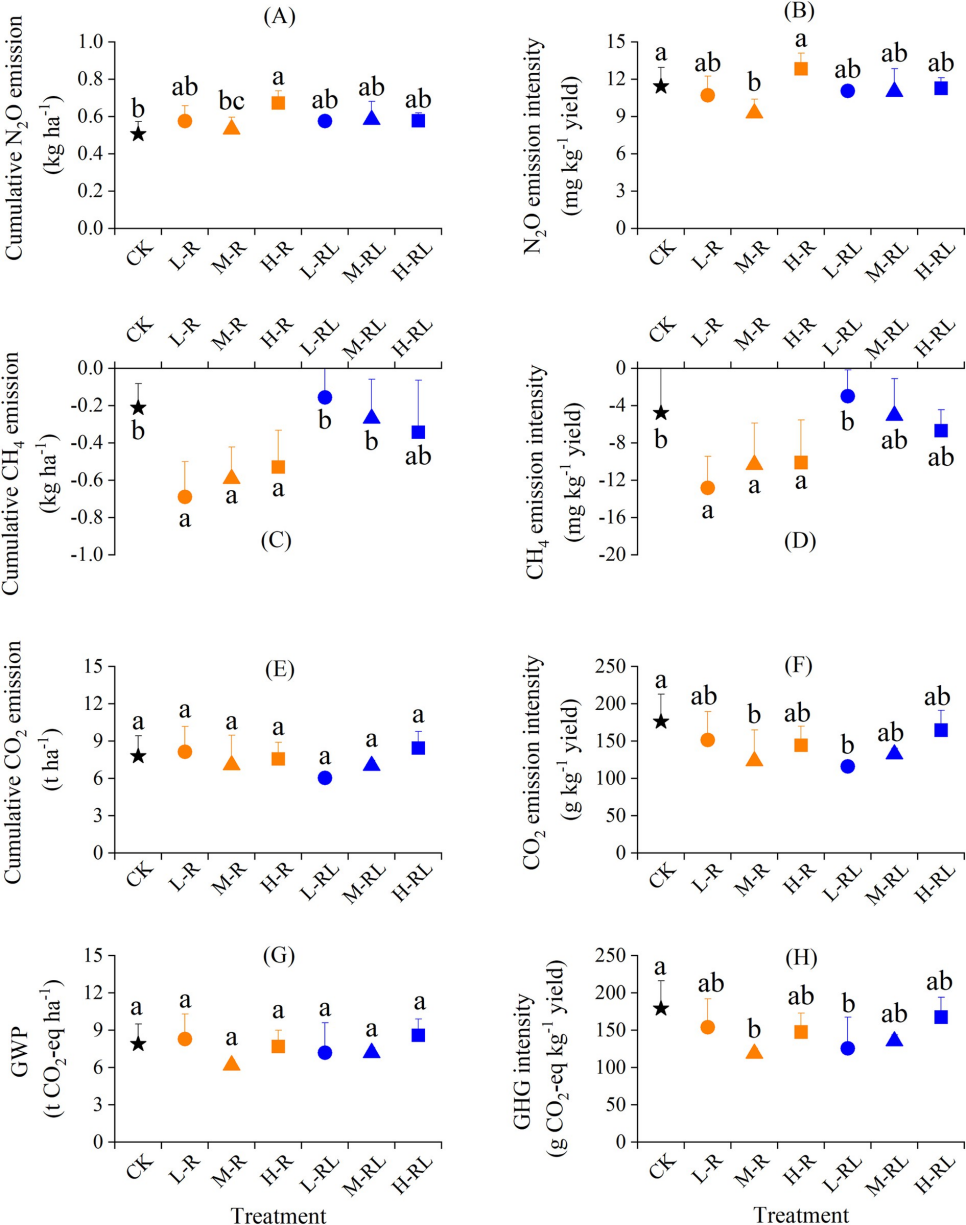
Cumulative emissions and emission intensity of N_2_O (A, B), CH_4_ (C, D) and CO_2_ (E, F) as well as GWP (G) and GHG intensity (H) in different treatments. Error bars indicate standard deviation of three replicates. Different lowercase letters indicate significant differences among treatments at *P* < 0.05.

The N_2_O fluxes were positive from all treatments, indicating that the hawthorn orchard soil was a net source of N_2_O (Fig 3A). The N_2_O fluxes, ranging from 0.9 to 22.6 g ha^−1^ d^−1^ with an average of 5.1 g ha^−1^ d^−1^, were significantly affected by treatment, sampling date and their interactions. Cumulative N_2_O emissions were 0.5−0.7 kg ha^−1^, and were not significantly enhanced by microalgae application except H-R treatment (Fig 4A). Moreover, cumulative N_2_O emissions in M-R treatment were significantly lower by 20.9% than in H-R treatment. As for N_2_O emission intensity, it was 9.3−12.8 mg kg^−1^ yield, and was significantly lower by 18.9% and 27.8% in M-R treatment than CK and H-R treatment, respectively (Fig 4B).

The CH_4_ fluxes ranged from −28.5 to 17.4 g ha^−1^ d^−1^ with an average of −4.3 g ha^−1^ d^−1^ (Fig 3B), and cumulative CH_4_ emissions was −0.7 to −0.2 kg ha^−1^ (Fig 4C). This indicated that the soil acted as a small sink for atmospheric CH_4_. Microalgae in root application significantly reduced the cumulative CH_4_ emissions by 1.5−2.3 folds, with the largest reduction in L-R treatment. A similar effect of microalgae application was also observed in CH_4_ emission intensity (Fig 4D).

Soil CO_2_ fluxes ranged from 20.1 to 160.4 kg ha^−1^ d^−1^ with an average of 62.0 kg ha^−1^ d^−1^, and were remarkedly affected by treatment and sampling date (Fig 3C). Cumulative CO_2_ emissions were 6.0−8.4 t ha^−1^, and not significantly influenced by microalgae application (Fig 4E). The CO_2_ emission intensity ranged between 116.0 and 176.2 g kg^−1^ yield, and were significantly lower by 29.9% and 34.2% in M-R and L-RL treatments than CK, respectively (Fig 4F).

The GWP, ranging between 6.2 and 8.6 t CO_2_-eq ha^−1^, was not significantly different among treatments (Fig 4G). While the GHG intensity, varying from 118.9 to 179.2 g CO_2_-eq kg^−1^ yield, decreased by 6.4%−33.6% as compared to CK, and M-R and L-RL treatments had higher and significant decreases (Fig 4H).

### Soil organic carbon fractions and soil pH

**Fig 5:**
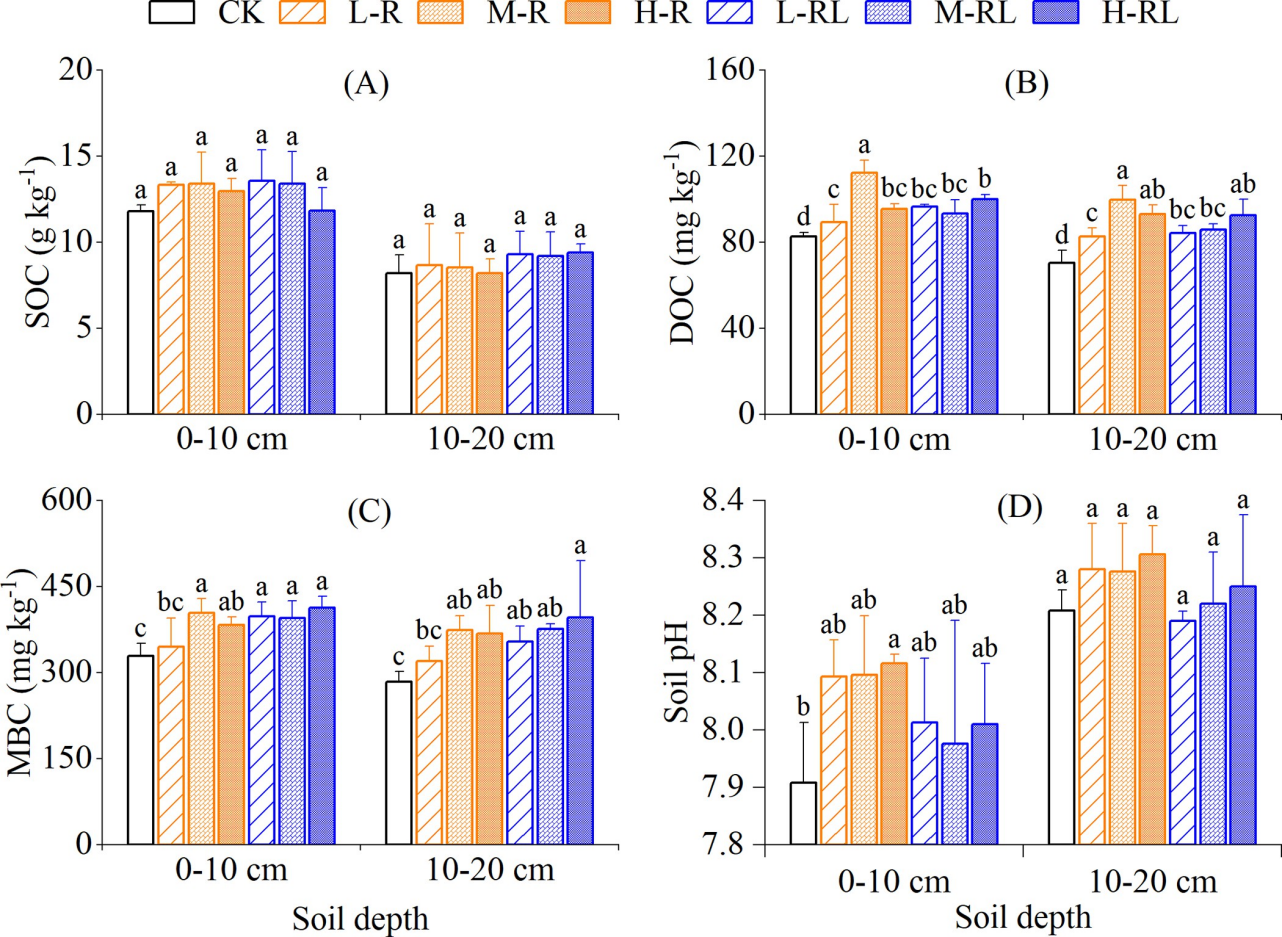
Contents of SOC (A), DOC (B) and MBC (C) as well as soil pH (D) at 0−10 cm and 10−20 cm soil depth in different treatments. Error bars indicate standard deviation of three replicates. Different lowercase letters indicate significant differences among treatments in each soil depth at *P*<0.05.

The SOC contents ranged from 11.8 to 13.6 g kg^−1^ and 8.2 to 9.4 g kg^−1^ in 0−10 cm and 10−20 cm, respectively, and was significantly higher in 0−10 cm than that in 10−20 cm for all the treatments (Fig 5A; Table 2, *P* < 0.05). Compared with CK, microalgae application increased SOC content by 4.1%−14.8% except for H-R treatment of 10−20 cm, although not significantly.

**Table 2:** ANOVA results (*P*-values) for the effects of treatment, soil depth and their interactions on soil carbon and nitrogen fractions as well as soil pH. Significant effects (*P* < 0.05) are highlighted in bold.

	df	SOC	DOC	MBC	NH_4_^+^−N	NO_3_^−^−N	DON	MBN	pH
Treatment	6	0.362	**0.000**	**0.000**	**0.021**	0.156	**0.044**	0.824	0.252
Soil depth	1	**0.000**	**0.000**	**0.013**	0.652	**0.000**	**0.005**	**0.029**	**0.000**
Treatment×Soil depth	6	0.682	0.417	0.962	0.735	0.390	0.852	0.908	0.953

Treatment and soil depth had strong effects on DOC and MBC contents, but their interaction effect was not observed (Table 2). Compared with CK, microalgae application significantly increased DOC content by 8.1%−35.9% and 19.5%−41.4% in 0−10 cm and 10−20 cm, respectively, with a maximal increment in M-R treatment that was significantly higher than other treatments (Fig 5B). Except for L-R treatment, microalgae application significantly increased MBC content by 16.1%−25.4% and 24.6%−39.2% in 0−10 cm and 10−20 cm as compared to CK, respectively, with a maximal increment in H-RL treatment (Fig 5C).

The soil pH increased with soil depth, ranging from 7.91−8.12 in 0−10 cm to 8.19−8.31 in 10−20 cm depth (Fig 5D; Table 2, *P* < 0.05). Compared with CK, H-R treatment markedly increased soil pH by 0.21 units in 0−10 cm depth. However, the difference was not significant among treatments in 10−20 cm depth.

### Soil nitrogen fractions

**Fig 6:**
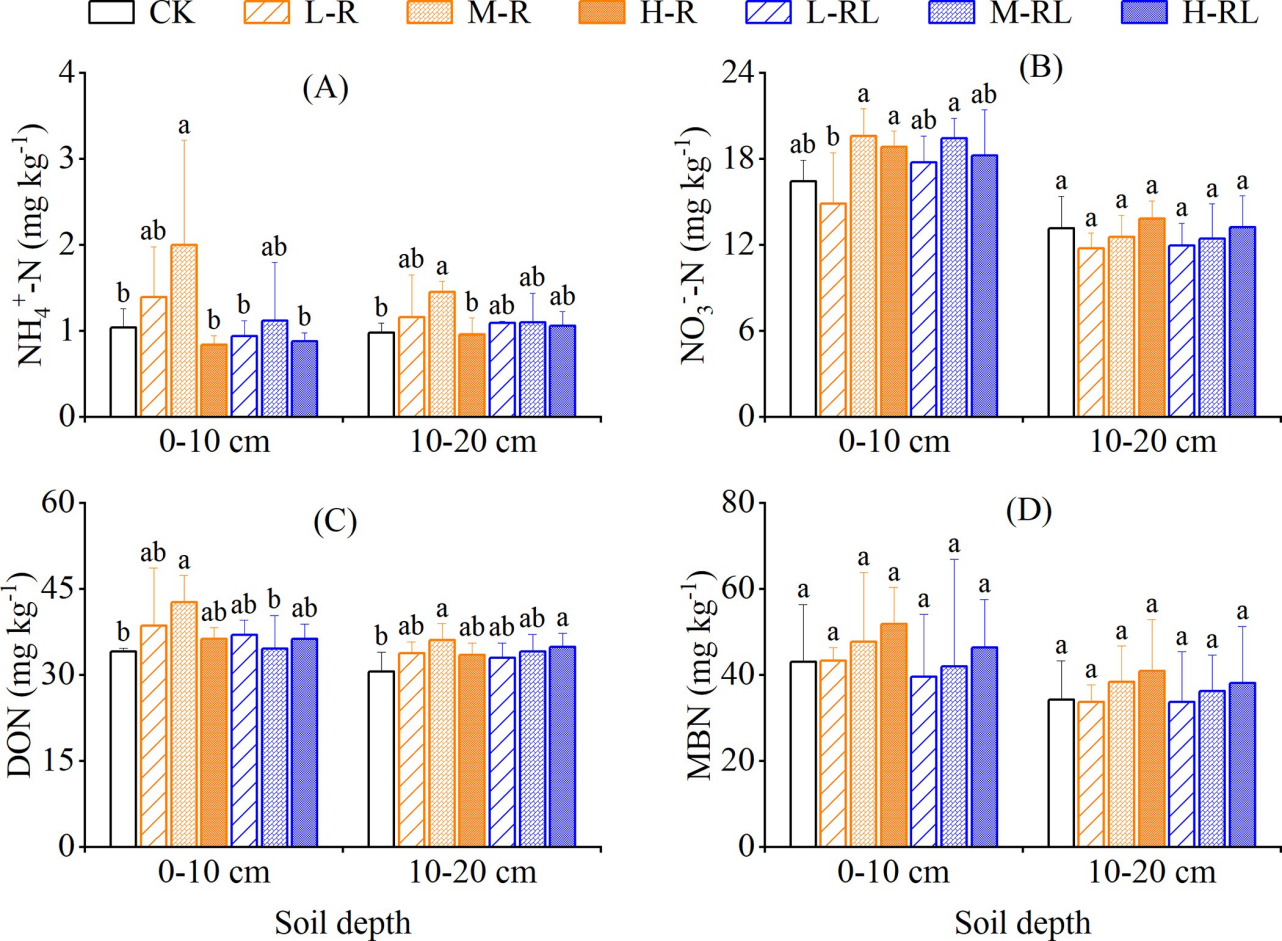
Contents of NH_4_^+^−N (A), NO_3_^−^−N (B), DON (C) and MBN (D) in 0−10 cm and 10−20 cm soil depth in different treatments. Error bars indicate standard deviation of three replicates. Different lowercase letters indicate significant differences among treatments in each soil depth at *P* < 0.05.

Treatments had strong effects on NH_4_^+^−N content (Table 2). Compared with CK, only M-R treatment significantly increased NH_4_^+^−N content by 92.0% and 48.1% in 0−10 cm and 10−20 cm, respectively, and was significantly higher than H-R treatment in both soil depth (Fig 6A). NO_3_^−^−N content was significantly higher in 0−10 cm than that in 10−20 cm for all the treatments, but there was no remarkable difference among treatments, except M-R, H-R and M-RL treatments in 0−10 cm depth that showed a significant increase compared with L-R treatment (Table 2, Fig 6B).

Both treatments and soil depth had strong effects on DON content (Table 2). Compared with CK, only M-R treatment significantly increased DON content by 25.2% and 17.9% in 0−10 cm and 10−20 cm, respectively, and was significantly higher than M-RL treatment in 0−10 cm depth (Fig 6C). As for MBN content, it was significantly influenced by soil depth, but there was no significant difference among treatments (Table 2, Fig 6D).

## Discussions

### Fruit productivity and quality

The yield of hawthorn fruit was significantly improved by microalgae application in relative to single conventional fertilization, with the largest increment in M-R treatment (Fig 2A). A number of previous studies have also reported the beneficial effects of microalgae-based biofertilizer on plant growth and yield [15, 17, 19, 47]. Microalgae can provide organic nutrients through photosynthesis and cell lysates, leading to stimulation of microbial activity that facilitated mineralization and mobilization of nutrients for plant growth [27]. In addition, after adding nitrogen-fixing cyanobacteria members, such as *Anabaena azotica* employed in this study, the enhancement of soil microbial activity accelerates soil nitrogen transformation and promoted nitrogen mineralization [14]. As a consequence, the breakdown of microalgae may lead to releasing various nutrients and increase soil mineralization [26], which was beneficial to the improvement of soil available nitrogen [48]. Our results showed that M-R treatment significantly increased soil NH_4_^+^−N and DON contents in 0−10 cm soil depth (Fig 6A and 6C). Soil DON was an important pool for nitrogen transformations and a potential source of plant nitrogen nutrition [49], because the main route to produce NH_4_^+^−N in soil was likely through extracellular enzymes that first convert insoluble organic nitrogen into soluble organic nitrogen [50]. This may reflect in a higher soil DON coincided with the higher NH_4_^+^−N and fruit yield in M-R treatment. On the other hand, microalgae had the ability to supply micronutrients (e.g. iron, manganese, copper and zinc) and phytohormones (e.g. auxins, gibberellins and cytokinins) [17, 51], which were related to plant growth and thus could enhance plant productivity. The significant increase in the contents of soluble and reducing sugars in H-RL treatment (Fig 2E and 2F), might be related to the abundant chlorophyll, phytohormone, and photosynthate resulting from microalgae proliferation [15, 52–54]. In addition, foliar spray of microalgae has been reported to improve the water use efficiency and stomatal functioning in plants [53]. Hajnal-Jafari et al. [28] reported that foliar spray of chlorella suspensions acted more excellently in enhancing the quality and yield of Swiss beet than root application of chlorella suspensions. However, in this study, there was no significant difference between root application and foliar spray at the same total microalgae doses regarding the hawthorn fruit yield and quality.

### Linking soil carbon and nitrogen fractions to GHG emissions

Various practices and technologies have been attempted to decrease N_2_O emissions from fruit orchards [6]. Microbiological technologies to mitigate soil N_2_O emissions have been achieved through inoculation with N_2_O-reducing denitrifiers to roots, soils or fertilizers [55, 56]. Our results showed that microalgae application did not increase N_2_O emissions from hawthorn orchard soil compared with conventional fertilization alone, except H-R treatment (Figs 3A and 4A). A recent study showed that N_2_O emissions were 1.5−3.0 times lower in wheat soils fertilized by green microalgae compared to inorganic fertilizer, and ascribed it mainly to lower soil inorganic nitrogen availability with microalgae application [32]. In this study, except for soil NH_4_^+^−N of M-R treatment, the microalgae application did not significantly change both NH_4_^+^−N and NO_3_^−^−N (Fig 6A and 6B). Unfortunately, we did not measure soil NH_4_^+^−N and NO_3_^−^−N at the peak of N_2_O emissions, due to logistical reasons. In contrast, the work of Castro et al. [33] showed a 5-time increase in N_2_O emissions under microalgal biofilm treatment compared with inorganic fertilizer, and attributed to larger labile organic carbon. Here, we indeed observed significant increases of soil labile carbon (i.e. DOC & MBC) in microalgae application (Fig 5B and 5C). Moreover, the effects of microalgae application on soil microbes responsible for N_2_O production may influence the observed N_2_O emissions. While Suleiman et al. [34] reported that no significant shift was found in the abundances of ammonia-oxidizing bacteria and archaea as well as denitrifying gene *nirS* and *nirK* in soils applied with microalgae. This suggests that the changes in soil microbes on biofertilizer may be temporary because of trend of the microbial community resilience [57]. In the present study with alkaline soil, H-R treatment resulted in an increase in N_2_O emissions compared with CK (Fig 4A), which could be partly ascribed to the higher soil pH in H-R treatment (Fig 5D). In alkaline agricultural soils under aerobic conditions, autotrophic nitrification was the dominant process for N_2_O production, and an increase in soil pH could stimulate autotrophic nitrification and N_2_O emissions [58, 59]. Additionally, live microalgae cells were likely able to synthesize N_2_O during their proliferation in the presence of available nitrogen source [60], and the microalgae-derived N_2_O production rates were linearly correlated to microalgal concentrations [61]. In this regard, the microalgae biofertilizer should consider and determine the appropriate dose to be used to minimize soil N_2_O emissions.

As the observed negative values of CH_4_ emissions (Fig 3B), the hawthorn orchard soil could act as an atmospheric CH_4_ sink, thanks to CH_4_ absorption or oxidation just like other well-drained upland soils [62, 63]. We observed that CH_4_ uptake was significantly increased by 1.5–2.3 times in soil with microalgae by root application as compared to single conventional fertilization (Fig 4C), which was inconsistent with the results of Castro et al. [33]. They showed that the presence of microalgal biofilm did not significantly influence CH_4_ emissions. Soil available carbon was an important substrate for CH_4_ production via methanogens [64]. The result of this study did observe significant increases in soil DOC with microalgae application (Fig 5B). However, not all the CH_4_ produced ends up in the atmosphere, because CH_4_ oxidizing bacteria (methanotrophs) is able to oxidize CH_4_ in the presence of oxygen. During microalgae photosynthesis, oxygen released can diffuse into the soil and result in aerobic soil microsites [65], which may decrease production and/or increase oxidation of CH_4_ especially in the presence of cyanobacteria [66]. Furthermore, previous studies found that CH_4_ oxidation was positively and significantly correlated with NO_3_^−^−N content [62, 67], because NO_3_^−^−N could promote the synthesis of enzymes involved in the CH_4_ oxidation process [68]. Therefore, we presumed that microalgae biofertilizer in root application may strengthen the population and activity of methanotrophs, and thus contributed to the higher CH_4_ uptake compared to conventional fertilization alone.

Regarding soil CO_2_ emissions, previous studies indicated that microalgae could induce greater microbial decomposition of organic matter or respiration with associated higher CO_2_ production [33, 34, 69]. In our study, microalgae application did not increase soil CO_2_ emissions (Fig 4E and 4F), although it did significantly increase soil microbial biomass (i.e. MBC) (Fig 5C). Considering that the live microalgae had the ability to consume CO_2_ via photosynthesis in the daytime [70], and might contribute to organic carbon accumulation through the proliferation of cells and the excretion of organic soil-binding metabolites [71, 72]. For example, Tu et al. [73] reported that the CO_2_ fixation rate of *Chlorella pyrenoidosa* was 1.2 g L^−1^ and it increased the dry biomass by around 85%. Additionally, earlier research stated that microalgae biofertilizer could result in a significant increase of SOC and a further increase when combined with organic fertilizer [21]. We also observed an increasing trend of SOC in the soils with microalgae biofertilizer (Fig 5A), albeit not significantly due to the short timescale of microalgae application in relative to SOC turnover [74]. It is anticipated that long-time microalgae application may retain more carbon in the soil through carbon sequestration process [23] and that may not yield an increase in CO_2_ emissions to atmosphere.

Taken together, our results showed that application of microalgae biofertilizer did not increase comprehensive greenhouse effect (i.e. GWP, Fig 4G). Additionally, linking hawthorn yield with GWP indicated that, microalgae application decreased GHG intensity and showed a significant decrease in M-R and L-RL treatments compared with CK (Fig 4H). The findings of this study suggested that applying microalgae could cause an increase in hawthorn yield and did not increase the GHG emissions cost, especially in M-R treatment.

## Conclusions

Our study showed that conventional fertilization combined with microalgae-based biofertilizer improved the hawthorn yield by 15.7%−29.6%, with a maximal increment at medium dose by root application. While microalgae application did not concomitantly increase the GHG emissions from hawthorn orchard soil in the view of GWP and GHG intensity. Apart from higher N_2_O at high dose by root application, microalgae significantly increased CH_4_ uptake by 1.5−2.3 times in root application. Moreover, microalgae application showed an increasing trend in SOC content, though not significantly due to the short timescale of the experiment, and significantly increased the labile organic carbon content. Overall, these results indicated that the medium microalgae dose with root application had a high fruit yield with low GHG emissions cost. The study highlighted the promise of the live microalgae as an effectively green biofertilizer, which could be expected to reduce GHG emissions in the context of lowering chemical fertilizer rates and remain fruit yield, recommending applying at medium microalgae dose with root application.
